# A Case of Premature Triple Vessel Coronary Artery Disease and Valvular Degeneration: A Rare Presentation of Familial Hypercholesterolemia

**DOI:** 10.7759/cureus.11037

**Published:** 2020-10-19

**Authors:** Rukham Gull, Muhammad Humayoun Rashid, Muhammad Saad Aleem, Muhammad Omar Salman, Ammar Khalid

**Affiliations:** 1 Internal Medicine, Nishtar Medical University, Multan, PAK; 2 Cardiology, Chaudhry Pervaiz Elahi Institute of Cardiology, Multan, PAK; 3 Orthopedics, Nishtar Medical University, Multan, PAK; 4 Medicine, Qamar Hospital Bagh AJK, Bagh AJK, PAK

**Keywords:** familial hypercholesterolemia, coronary artery disease, valvular degeneration, ct agiography, non-st segment elevation myocardial infarction (nstemi), ldl cholesterol, echocardiography

## Abstract

Familial hypercholesterolemia (FH) is one of the inherited causes of coronary artery disease (CAD) and causes calcific valvular degeneration in rare cases. A 13-year-old boy with multiple xanthomas presented with severe chest pain, shortness of breath, and sweating. He was diagnosed with premature CAD leading to non-ST-elevation myocardial infarction, secondary to early-onset FH [severely raised low-density lipoprotein (LDL) and triglycerides (TG) on lipid profile]. CT angiogram showed triple vessel disease, and echocardiogram revealed tight aortic stenosis. Percutaneous coronary angioplasty was done, and valvuloplasty was planned on the follow-up assessment. Early diagnosis and prompt management could have prevented these complications.

## Introduction

Familial hypercholesterolemia (FH) is a common familial cause of coronary artery disease (CAD) and premature death. It is a genetic disorder of lipoprotein metabolism caused by mutations in the low-density lipoprotein (LDL) receptor, characterized by very high plasma concentrations of low-density lipoprotein cholesterol (LDL-C), tendon xanthomas, and increased risk of premature CAD [1]. Decreased clearance of LDL-C leads to deposition in various tissues such as tendons, skin, and eyelids. It can also cause endothelial dysfunction by forming atheromatous plaques and can lead to vascular disorders such as CAD.

FH occurs in two forms: heterozygous and homozygous. Heterozygous FH is more prevalent, occurring in approximately 1/500, whereas, homozygous FH is rare (occurring in approximately 1/1,000,000) [2]. FH has been an excellent model of hypercholesterolemia as a powerful risk factor for CAD. Although it is commonly accepted that hypercholesterolemia is a coronary-oriented risk factor in both the FH and non-FH populations [3-5], what is of more importance is that aortic valvular dysfunction is a highly frequent and pivotal disorder in many FH cases and that, for some patients, is a life-threatening complication. Likewise, this case describes a patient with FH causing CAD leading to acute myocardial infarction along with severe aortic valvular stenosis, which led to left ventricular dysfunction.

## Case presentation

A 13-year-old obese boy presented to the cardiology emergency department with concerns of shortness of breath, sweating, and chest pain radiating to both shoulders and neck. His father reported that the patient had been experiencing shortness of breath on minimal exertion for the past two weeks. An electrocardiogram (ECG) showed T-wave inversions in V3 to V6, I, and augmented vector left (aVL), and mild ST-segment depressions in V4 and V5. His cardiac enzymes were raised, and troponin I was positive. He was started on dual antiplatelet therapy, heparin infusion, antianginal drugs, and oxygen as needed. He had a significant family history of hypercholesterolemia. His father had been on treatment for high cholesterol levels (350 mg/dl), whereas his grandfather had suffered a sudden cardiac death due to premature CAD.

**Figure 1:**
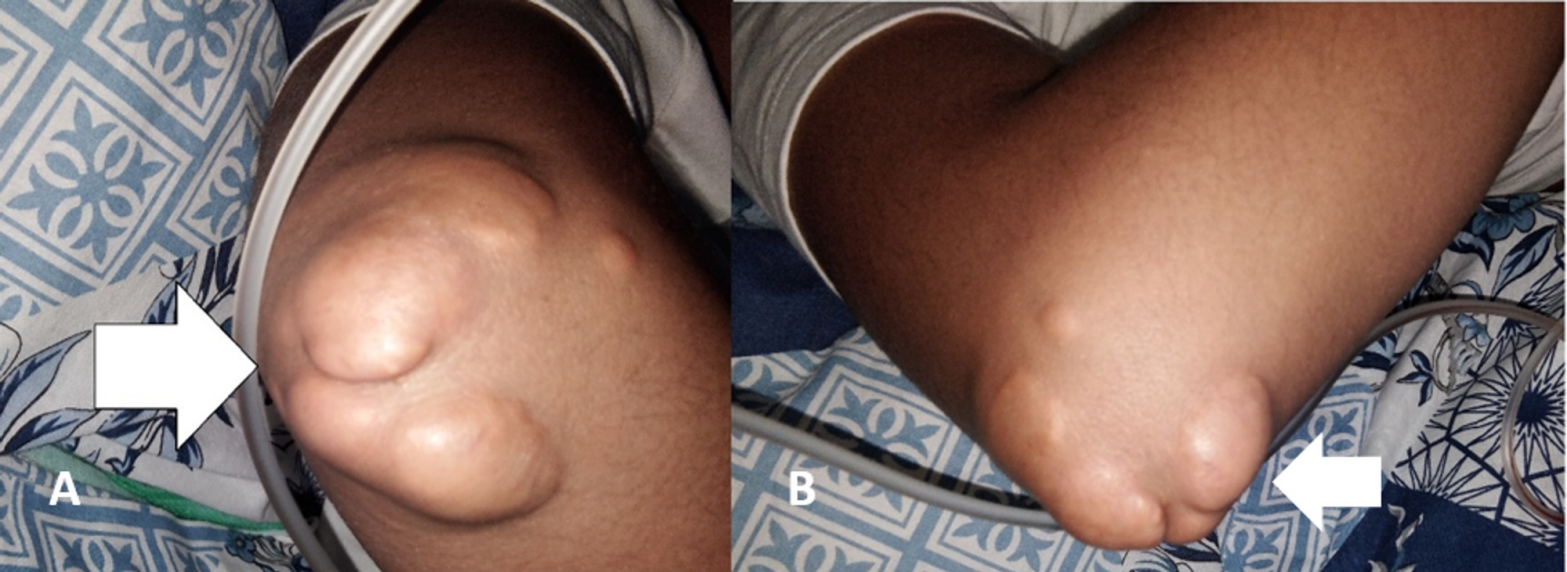
Tendon xanthomas (A, B) in the elbow region

On examination, the patient’s weight was 45 kg, height was 123 cm, and his body mass index (BMI) was 29.7 kg/m^2^. His vitals were as follows: blood pressure of 90/60 mmHg; pulse rate of 90 beats per minute; respiratory rate of 28 per minute; and temperature of 98 °F. There were obvious xanthomas on the right elbow, as shown in Figure 1, and minor ones on the shoulder and ankles. On auscultation, normal S1 and soft S2 with reverse splitting were present. A systolic ejection murmur was heard over the right second intercostal space, of mild intensity, radiating towards the carotids, and not audible without a stethoscope. No thrush was felt on palpation. It was assumed as a Grade 1 murmur of aortic stenosis.

**Figure 2:**
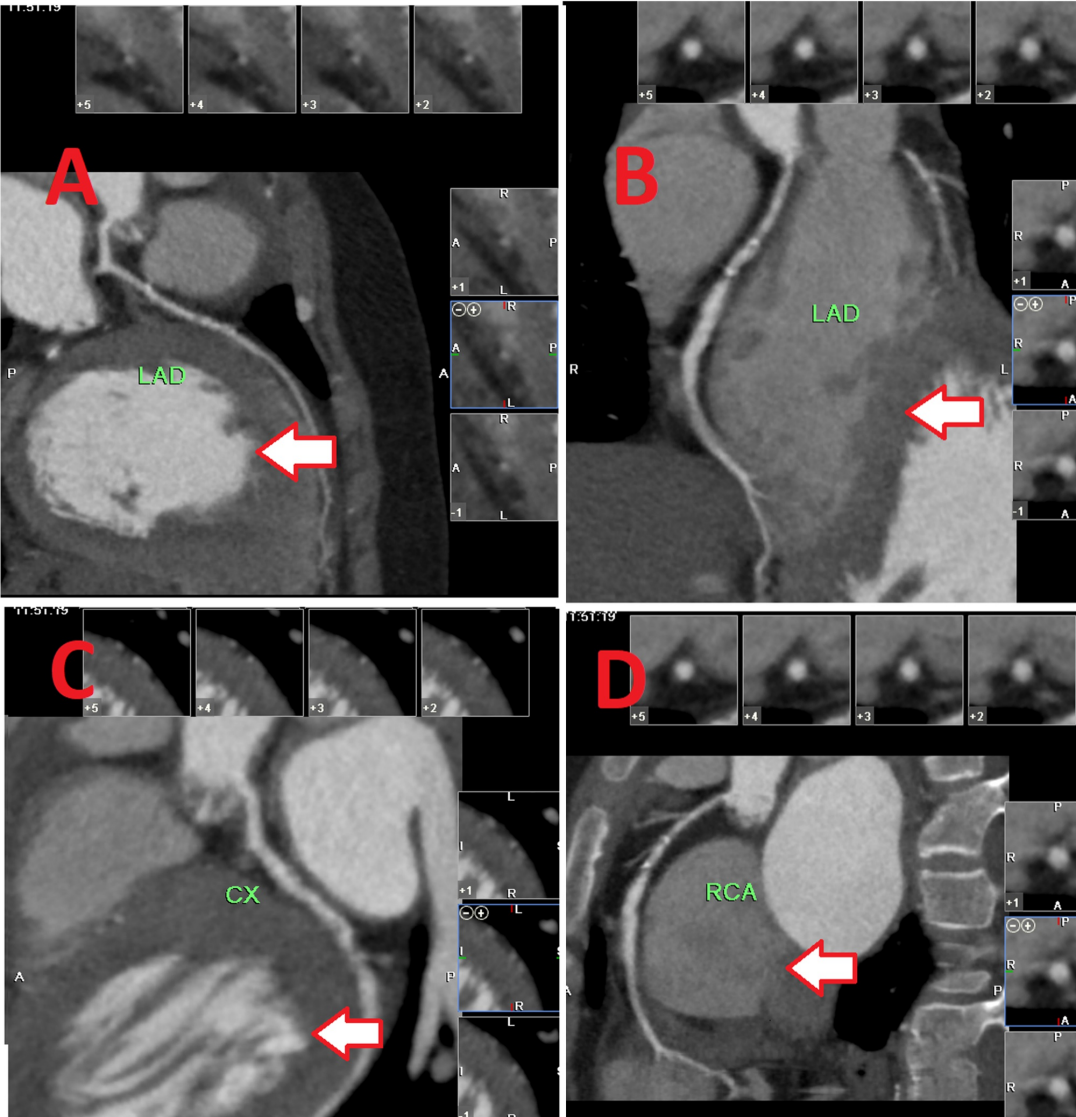
CT coronary angiogram A) left anterior descending artery (horizontal section) with moderately severe mid disease; B) left anterior descending artery (vertical section) with moderately severe mid disease; C) left circumflex artery with mid severe disease; D) right coronary artery with mild ostio-proximal disease CT: computed tomography

The patient remained admitted for four days, during which further investigations were done. Baseline investigations, glycated hemoglobin (HbA1c), and lipid profiles were performed. The lipid profile was as follows: total cholesterol: 822 mg/dl; LDL: 790 mg/dl; high-density lipoprotein (HDL): 32 mg/dl; triglycerides (TG): 175 mg/dl; non-HDL cholesterol: 790 mg/dl. He was diagnosed with FH. Echocardiography revealed bicuspid aortic valve and degenerative calcifications leading to severe aortic stenosis with an aortic valve area of 0.5 cm^2^, stroke volume of 35 ml/beat, and diffuse left ventricular hypertrophy with left ventricular ejection fraction of 30%. CT coronary angiography was done, as shown in Figure 2.

The CT angiogram revealed a good-sized left anterior descending (LAD) artery with a moderately severe mid disease (Figures 2A, 2B), a non-dominant left circumflex artery (LCX) artery with severe mid disease (Figure 2C), and a dominant right coronary artery (RCA) with mild ostio-proximal disease (Figure 2D). Calcium scores were as follows: LAD: 34; left circumflex artery (LCX) 30; right coronary artery (RCA): 2; and a total score of 66. Percutaneous coronary angioplasty was done in the LCX and LAD. 3D views of the patient's CT coronary angiography are shown in Video 1 and Video 2.

**Video 1:** 3D view of CT coronary angiography 1 CT: computed tomography

**Video 2:** 3D view of CT coronary angiography 2 CT: computed tomography

The patient was put on dual antiplatelet therapy (DAPT), consisting of aspirin 75 mg once-daily (OD) and clopidogrel 75 mg OD, and high-dose statin therapy. Furthermore, aortic valve replacement was also planned on the follow-up to preserve left ventricular function. Lifestyle and dietary modifications were recommended. He was booked in for a one-month follow-up for clinical and biochemical assessment.

## Discussion

Diagnosis of FH depends upon physical examination findings, laboratory results, and family history. Furthermore, molecular and genetic diagnostic methods are also used where the facilities are available. If genetic testing confirms the diagnosis, other family members can be advised to get tested as well. The Dutch Lipid Clinic Network Diagnostic Criteria for FH was used in this case, and it considers the LDL-C levels, clinical signs, and family history [6]. Our patient presented with signs of acute coronary syndrome, which was diagnosed as a non-ST-elevation myocardial infarction based on positive ECG and lab findings. This unusual presentation, coupled with a family history of hypercholesterolemia, mandated further investigations, including a lipid profile, which showed raised TG, LDL-C, and non-HDL-C.

Symptoms certainly vary between the homozygous and heterozygous FH. Patients with homozygous FH may have symptoms of ischemic heart disease, peripheral vascular disease, cerebrovascular disease, and valvular degeneration. Tendonitis and arthralgias can also be the presenting symptoms. In such circumstances, a high level of suspicion is necessary to diagnose FH. Heterozygous FH patients usually do not have such severe symptoms. They usually present with interdigital planar xanthomas, cutaneous xanthomas, tendon xanthomas, and familial clustering. One of their parents will have severe hypercholesterolemia, and 50% of the patient’s siblings will also be heterozygous because the gene for FH is dominant [7].

Our patient presented with early-onset CAD confirmed by CT coronary angiogram, which showed triple vessel disease. This CAD leads to ischemic heart disease, as confirmed by ECG changes and raised troponins. He also had severe aortic stenosis, low-flow, low-gradient, as confirmed by transthoracic echocardiography. His ejection fraction measured by a coronary angiogram was 30%, secondary to ischemic heart disease and aortic stenosis. A diagnosis of FH (probably homozygous) was made as his genetic testing could not be done at that moment due to limited resources but was planned for the future. A fasting lipid profile of all his family members was also planned. For severely damaged aortic valves, valvuloplasty can be done.

FH cannot be treated by diet and exercise alone. These lifestyle changes can help, but medications are required when LDL-C levels need to be reduced significantly (as much as by 50% or 75%). Treatment usually involves statin drugs, ezetimibe, and bile acid sequestrants, alone or in combination. People with extremely high LDL, like those with homozygous FH, may need to undergo a treatment called LDL apheresis. This is a dialysis-like procedure that is done every few weeks to remove cholesterol from the blood [8]. Newer treatment options are also being studied.

## Conclusions

We discussed the case of a 13-year-old boy who presented with multiple xanthomas, severe chest pain, shortness of breath, and sweating and was subsequently diagnosed with premature CAD leading to non-ST-elevation myocardial infarction, secondary to early-onset FH. FH can lead to severe endothelial damage, atherosclerosis, early-onset CAD, and degenerative changes in heart valves. A high index of suspicion is required for early diagnosis and prompt treatment. This can help prevent a potentially life-threatening complication, such as CAD and sudden cardiac death.

## References

[1] Nordestgaard BG, Chapman MJ, Humphries SE (2013). Familial hypercholesterolaemia is underdiagnosed and undertreated in the general population: guidance for clinicians to prevent coronary heart disease: consensus statement of the European Atherosclerosis Society. Eur Heart J.

[2] Rader DJ, Cohen J, Hobbs HH (2003). Monogenic hypercholesterolemia: new insights in pathogenesis and treatment. J Clin Invest.

[3] Stone NJ, Levy RI, Fredrickson DS, Verter J (1974). Coronary artery disease in 116 kindred with familial type II hyperlipoproteinemia. Circulation.

[4] Jensen J, Blankenhorn DH, Kornerup V (1967). Coronary disease in familial hypercholesterolemia. Circulation.

[5] Stamler J, Wentworth D, Neaton JD (1986). Is relationship between serum cholesterol and risk of premature death from coronary heart disease continuous and graded? Findings in 356,222 primary screenees of the Multiple Risk Factor Intervention Trial (MRFIT). JAMA.

[6] Al-Rasadi K, Al-Waili K, Al-Sabti HA, Al-Hinai A, Al-Hashmi K, Al-Zakwani I, Banerjee Y (2014). Criteria for diagnosis of familial hypercholesterolemia: a comprehensive analysis of the different guidelines, appraising their suitability in the Omani Arab population. Oman Med J.

[7] Klose G, Laufs U, März W, Windler E (2014). Familial hypercholesterolemia: developments in diagnosis and treatment. Dtsch Arztebl Int.

[8] Raal FJ, Hovingh GK, Catapano AL (2018). Familial hypercholesterolemia treatments: guidelines and new therapies. Atherosclerosis.

